# Probabilistic Diffusion Tractography Reveals Improvement of Structural Network in Musicians

**DOI:** 10.1371/journal.pone.0105508

**Published:** 2014-08-26

**Authors:** Jianfu Li, Cheng Luo, Yueheng Peng, Qiankun Xie, Jinnan Gong, Li Dong, Yongxiu Lai, Hong Li, Dezhong Yao

**Affiliations:** 1 Key Laboratory for NeuroInformation of Ministry of Education, School of Life Science and Technology, University of Electronic Science and Technology of China, Chengdu, China; 2 Key Laboratory of Cognition and Personality of Ministry of Education, Southwest University, Chongqing, China; Beijing Normal University, China

## Abstract

**Purpose:**

Musicians experience a large amount of information transfer and integration of complex sensory, motor, and auditory processes when training and playing musical instruments. Therefore, musicians are a useful model in which to investigate neural adaptations in the brain.

**Methods:**

Here, based on diffusion-weighted imaging, probabilistic tractography was used to determine the architecture of white matter anatomical networks in musicians and non-musicians. Furthermore, the features of the white matter networks were analyzed using graph theory.

**Results:**

Small-world properties of the white matter network were observed in both groups. Compared with non-musicians, the musicians exhibited significantly increased connectivity strength in the left and right supplementary motor areas, the left calcarine fissure and surrounding cortex and the right caudate nucleus, as well as a significantly larger weighted clustering coefficient in the right olfactory cortex, the left medial superior frontal gyrus, the right gyrus rectus, the left lingual gyrus, the left supramarginal gyrus, and the right pallidum. Furthermore, there were differences in the node betweenness centrality in several regions. However, no significant differences in topological properties were observed at a global level.

**Conclusions:**

We illustrated preliminary findings to extend the network level understanding of white matter plasticity in musicians who have had long-term musical training. These structural, network-based findings may indicate that musicians have enhanced information transmission efficiencies in local white matter networks that are related to musical training.

## Introduction

As is widely known, musicians represent an ideal model to investigate experience-driven plasticity changes in the human brain related to their long-term complex musical training and performances [1], [2]. In the past ten years, many researchers have focused on the neuroplasticity of musicians' brains using diverse technologies and methods, including functional magnetic resonance imaging (fMRI), electroencephalography (EEG), magnetoencephalography (MEG), diffusion-weighted imaging (DWI), voxel-based morphometry (VBM), and surface-based morphometry (SBM).

In fMRI studies, musicians have exhibited different activation patterns during specific tasks. For example, musicians displayed increased activation of the premotor, primary, and supplementary motor cortices during motor or auditory tasks [3], [4], [5]; enhanced activities in the left middle and superior temporal gyri, the left inferior frontal gyrus, and the right ventromedial prefrontal cortex in response to pattern deviation [6]; greater activation in the bilateral visual cortex during memory retrieval [7]; and unique activities in the auditory association cortex during conceptual processing of visually presented musical instruments [8]. Based on EEG data, researchers showed that musicians exhibit enhanced event-related brain potentials in response to sound omissions and auditory-evoked potentials during specific auditory tasks by analyzing the mismatch negativity (MMN) component [9] and the P2 and N1 components separately [10], [11]. MEG studies have shown increased somatosensory representation of the fingers of the left hand in string players compared to control group [12], as well as an increased auditory cortical representation in musicians versus non-musicians [13], [14]; additionally, the locations of the equivalent current dipoles (ECDs) for the noise burst were significantly posterior to the ECDs for the tones in the two hemispheres of the musicians, but were not in the non-musicians [15]. These event-related functional imaging results suggested that musicians had better performances in specific sensory, motor, and auditory tasks. Musical training may have triggered intrinsic plasticity in the corresponding cortices. Other fMRI studies have also observed reduced activation of primary motor cortical areas in musicians, they deduced that possibly due to fewer activated neurons showing increased efficiency [16], [17]. Moreover, in our previous resting-state fMRI study, enhanced integration of the motor and perceptual systems was observed in musicians [18]. In summary, these findings demonstrated that intensive musical experience could induce changes in functional plasticity in the human brain and may increase the efficiency of integration and the processing of motor, auditory, and visual information.

VBM, SBM and DWI-based approaches have been used to study anatomical plasticity. VBM studies have resulted in some evidence of structural differences in the gray matter of musicians and non-musicians. Researchers found that these differences were distributed in several regions in the brain, including the anterior corpus callosum [19], the planum temporale [20], [21], the primary hand motor area, the cerebellum [22], [23], [24], Broca's area [25], the right auditory cortex [26], and Heschl's gyrus [27]. Recently, using SBM, Li et al. [28] found that musicians showed greater local variability in the middle section (i.e., somatotopic hand area) of the right central sulcus (CS) and the lower section of the left CS compared to the controls. In conclusion, studies focused on gray matter features have shown that intensive musical experience may result in the increased volume or local variability of the surface of gray matter regions related to musical training.

Taken together, these functional and structural studies found that musicians had different neuronal activation patterns and/or anatomical features in some sensory-, motor-, auditory-, and visual-related brain regions in comparison with non-musicians. These findings may reflect the stronger functional demands of these regions during musical training and performance and lend support to the hypothesis that training induces plasticity in these regions.

In contrast to the gray matter, the few studies have investigated the architectural changes in the white matter (WM) in response to musical training. The regions with group differences include the corpus callosum (body, genu, and splenium), the posterior limb of the internal capsule, and the right superior longitudinal fasciculus, which showed altered diffusion parameters, such as fractional anisotropy (FA) and diffusivity, in the WM of musicians versus non-musicians [29], [30]. Imfeld and colleagues observed that the FA values of musicians were bilaterally lower than those of non-musicians in the corticospinal tract, and diffusivity was negatively correlated with the onset of musical training in childhood [31]. Recently, using diffusion tensor tractography, an increased volume and number of streamlines of WM were observed in the right cerebellum in musicians [32]. These findings showed that musical training induced plasticity in the WM fibers that convey sensory and motor information.

The human brain is a complex network using multiple scales of time and space [33]. Numerous studies have explored the functional and structural networks in the human brain using complex network approaches and, in recent years, graph theory. Within graph analysis, the brain is modeled as a graph comprising *N* nodes connected by *M* edges. Based on the graph, through a rich set of mathematical tools and theoretical concepts, we can understand brain network more integrally. DWI tractography is a useful method that can be used to map human WM networks [34], [35], [36], [37], [38]. Gong and colleagues [39] showed that human WM structural networks showed prominent “small-world” attributes with embedded pivotal regions and exponentially truncated power-law topological distribution in long-range WM tracts. The small world topology could reveal the efficiency of a brain and play a central role in cortical information processing [40]. Many researches about neurological and psychiatric disorders showed that the small world efficiency of brain are changed in those patients with different kinds of diseases [41], [42]. Although many studies have demonstrated that long-term musical training can induce functional and structural changes in the brain, the training-induced influences on the WM network are still poorly understood. As far as we know, there is no study reporting alterations in the topological organization of the WM networks in musicians. Musical training could cause changes in WM plasticity, which are likely to be reflected in changes in the microstructure; therefore, changes in plasticity may also lead to changes in the topological organization of the WM networks. To investigate if and how the topological properties change, probabilistic diffusion tractography and graph theoretical approaches were used to analyze both musicians and controls.

## Materials and Methods

### Subjects

The subjects included 16 female musicians [range of age: 20–26 years; mean age: 23.3±2.5 years; mean duration of musical experience: 13.3±4.5 years (range of training duration: 6–20 years)] and 16 age- and gender-matched non-musicians (range of age: 20–25 years; mean age: 21.5±1.6 years). All of the participants were right-handed and were not diagnosed with any neurological and psychiatric disorders.

All musicians were students at the College of Music in the University of Southwest of China and majored in music. All musicians had received long-term training. Fourteen of musicians had piano training; meanwhile, ten of them also received variable degrees of training in other instruments (e.g., Chinese zither, accordion). The remained two musicians were major in the Chinese zither. Until the experiment began, musicians were continuously trained with several hours per day (1 hour in 5 subjects; 2 hours in 6 subjects; more than 3 hours in 5 subjects). All of the non-musicians reported that they had never received formal musical training or played any musical instrument. All of the subjects were recruited through advertisements at the Southwest University in China. The education years are not significant difference between the two groups (P = 0.11, T = 1.68). Informed written consent was obtained from all participants. The experimental protocol and informed consent were approved by the research ethics review board of the Southwest University in China.

### Image acquisition

For each subject, DWI and high-resolution T1-weighted structural MRI of the whole brain were acquired using a 3T Siemens Trio Tim MRI scanner (Siemens, Erlangen, Germany) with an eight-channel-phased array head coil in the Key Laboratory of Cognition and Personality of Ministry of Education, Southwest University, China.

Each DWI dataset consisted of a *b* = 0 image and 20 diffusion-weighted images with the following parameters: 50 slices of 2.5 mm thickness, with 3.25 mm between adjacent slices; *b* = 1000 s/mm^2^ for the weighted images; FOV = 220×220 mm^2^; acquisition matrix = 128×128, corresponding to an ‘in plane’ spatial resolution of 1.72×1.72 mm^2^; TE/TR = 104 ms/7200 ms; and a flip angle = 90°. The aforementioned acquisition was repeated three times to improve the reliability of data.

For the 3D T1-weighted images, the following scan parameters were used: 176 contiguous slices of 1 mm thickness in the sagittal orientation; in plane FOV = 224×256 mm^2^, with a spatial resolution of 1×1 mm^2^; TE/TR = 3.02 ms/2600 ms; and a flip angle = 8°.

### Preprocessing of DWI

A head-motion sketch of each DWI dataset was drawn by the Statistical Parametric Mapping software (SPM5, http://www.fil.ion.ucl.ac.uk/spm), and this procedure was, respectively, acted on three repeated acquisitions of each subject and datasets with excessive head-motion (translation >1 mm or rotation >1°) were excluded. To reduce the influence of head motion between repeated scans to fiber tracking, the remaining data sets for a given subject were not averaged before tensor calculation, but were processed by the following steps respectively. Preprocessing of the diffusion images of every subject was performed using the FSL software (FSL4.1.6, http://www.fmrib.ox.ac.uk/fsl) and consisted of brain extraction and eddy current correction. Based on corrected b-matrix [43] and images, the processes in FSL later will be executed.

### Segmenting and Registering

There are two steps to transform standard AAL [44] mask to native b0-space using the IBASPM toolbox (IBASPM, http://www.fil.ion.ucl.ac.uk/spm/ext/#IBASPM). First, the T1-weighted 3D anatomical image was normalized to standard AAL-space. Then the native T1-space AAL mask image was obtained through the inverse transformation. Second, the T1-weighted image was registered to the b0 diffusion image. T1-space AAL mask was transformed to b0-space in the same way. Hereto, we obtained the atlas image in b0-space including 90 gray matter structures, with 45 regions for each hemisphere (Table 1).

**Table 1:** Cortical and sub-cortical regions defined in Automated Anatomical Labeling template image in standard stereotaxic space.

Region name	Abbreviation	Region name	Abbreviation
Superior frontal gyrus, dorsolateral	SFGdor	Superior parietal gyrus	SPG
Superior frontal gyrus, orbital	SFGorb	Paracentral lobule	PCL
Superior frontal gyrus, medial	SFGmed	Postcentral gyrus	PoCG
Superior frontal gyrus, medial orbital	SFGmorb	Inferior parietal gyrus	IPG
Middle frontal gyrus	MFG	Supramarginal gyrus	SMG
Middle frontal gyrus, orbital	MFGorb	Angular gyrus	ANG
Inferior frontal gyrus, opercular	IFGoper	Precuneus	PCNU
Inferior frontal gyrus, triangular	IFGtri	Posterior cingulate gyrus	PCC
Inferior frontal gyrus, orbital	IFGorb	Insula	INS
Gyrus rectus	REG	Thalamus	THA
Anterior cingulate gyrus	ACC	Superior temporal gyrus	STG
Olfactory cortex	OLF	Superior temporal gyrus, temporal pole	STGp
Precentral gyrus	PreCG	Middle temporal gyrus	MTG
Supplementary motor area	SMA	Middle temporal gyrus, temporal pole	MTGp
Rolandic operculum	ROL	Inferior temporal gyrus	ITG
Median- and para-cingulate gyrus	MCC	Heschl gyrus	HES
Calcarine fissure and surrounding cortex	CAL	Hippocampus	HIP
Cuneus	CUN	Parahippocampal gyrus	PHIP
Lingual gyrus	LING	Amygdala	AMYG
Superior occipital gyrus	SOG	Caudate nucleus	CAU
Middle occipital gyrus	MOG	Lenticular nucleus, putamen	PUT
Inferior occipital gyrus	IOG	Lenticular nucleus, pallidum	PAL
Fusiform gyrus	FG		

### Fiber tracking and mapping zone-zone brain anatomical connections

To map the connections between the brain regions that were previously shown to be involved, we took three steps using the FSL software. First, the diffusion parameters were estimated to build up the distributions of the diffusion parameters at each voxel. Second, probabilistic tracking was performed on each mask. Finally, the connectivity matrix was established by taking the sum of the connection weight from region A to region B and that from region B to region A as the weight of the edge between regions A and B. Here, the connection weight from A to B is the number of pathways from A to B that were previously obtained divided by the number of voxels that are included in region A. Thus, the weight of the edge in the matrix could indirectly reflect the WM connectivity strength between two regions, and this matrix is symmetric. Self-connections were excluded. Hereto, one to three matrices corresponding to remained datasets of each subject were obtained. Finally, all of the connection matrices for each individual were averaged and this mean connection matrices were defined as the final network of the subject.

For further graph analysis, the connectivity backbones of each network were then estimated [45]. First, a maximum spanning tree, which connects all nodes of the network such that the sum of its weights is maximal, was extracted; then, additional edges were added in descending order of their weights until the average node degree was K. To ensure the sparseness and efficiency of the network, K was set as 4 on the basis of our previous experience [46] and another previous study (Hagmann et al., 2008). All posterior network analysis and visual representations were based on the resultant networks (connectivity backbones).

### Graph analysis of connection matrix

An *N*×*N* (*N* = 90 in the present study) weighted graph, *G* ^wei^, consisted of nodes (brain regions) and undirected edges. The weight of the edge from the *i*th node and the *j*th node was represented by 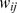. The subgraph *G_i_* was defined as the set of nodes that are direct neighbors of the *i*th node (i.e., directly connected to the *i*th node by an edge). *K_i_*, with *i* = 1, 2, …, 90 representing the degree of each node, was defined as the number of nodes in the subgraph *G_i_*. The strength, *S_i_*, was defined as the sum of the weights of the links connected to the *i*th node:
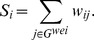
In this way, the degree and strength of a given node measured the extent to which the node was connected to the rest of the network.

Based on a previous study [47], 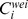 was adopted to quantify the clustering coefficient of the *i*th node in the weighted graph. This parameter shows how closely the nodes neighbored by the *i*th node are connected to each other and is calculated using the following equation:
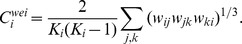



The clustering coefficient of a network is the average of the clustering coefficients of all nodes:
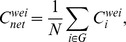
where 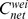 is a measure of the extent of the local density or cliquishness of the network.

Considering our use of the probabilistic tractography method, using the maximal weight of a network to normalize all of the weights might reduce the robustness. In this study, the original weights of each edge, but not the normalized ones, were used to calculate the clustering coefficient. Thus, we obtained the ‘generalized clustering coefficient’, which only indicates differences between two groups rather than the absolute quantity.

The shortest path length between the *i*th node and the *j*th node, labeled as 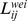, is the minimum distance of all paths between them. Path length is the sum of the direct distances of every pair of two neighbor nodes in the path; in this study, we choose the inversion of weight as the distance between two nodes. The mean shortest path length of a node was calculated using the following equation:
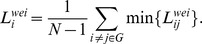



The characteristic path length of a network was measured by determining the ‘harmonic mean’ length between pairs [48] to overcome the problem of possibly disconnected network components. Formally, it is the reciprocal of the average of the reciprocals:
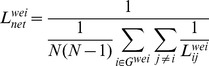
where 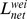 is a measure of the extent of average connectivity or overall routing efficiency of the network.

The nodal betweenness centrality, *BC_i_*, is the fraction of all of the shortest paths in the network that contain a given node:
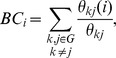
where 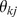 is the total number of shortest paths from the *k*th node to the *j*th node, and 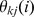 is the number of these paths passing through the *i*th node.

Nodes with high betweenness centrality values participate in a large number of the shortest paths.

The small-world attribute was originally proposed using the clustering coefficient and the shortest path length [49]. Compared to random networks, small-world networks typically have similar path lengths but higher clustering coefficients. In this study, we used network efficiency measures [50] to quantify the small-world behavior of the WM networks. These measurements included the global efficiency (*E_glob_*) and the local efficiency (*E_loc_*):
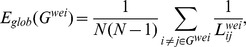


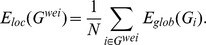
Practically, a network can be categorized as a small-world network if *E_glob_* is slightly less than and *E_loc_* is much greater than the matched random networks. For comparison purposes, we generated 100 random networks for each network using a Markov-chain algorithm [40], [51]; all 100 of these randomly generated networks were averaged to obtain a mean *E_glob_* and a mean *E_loc_* for each network. Typically, a small-world network *G* should meet the following criteria:
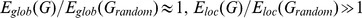



### Computation of network attributes and statistical analysis

Based on the formulas mentioned above and using the BCT toolbox [52], the individual network metric was calculated for each subject. To determine the differences in the topological organization of the WM networks between both groups, statistical comparisons of the features from local regions and the global network were performed using a two-sample two-tailed t-test. In detail, the features of the local regions included strength, degree, clustering coefficient and betweenness centrality of each region, and the global network features were represented by three small-world attributes (*E_loc_(G)/E_loc_(G_random_)* and *E_glob_(G)/E_glob_(G_random_)*).

## Results

All subjects succeeded in DWI scanning. According to the comparison of motion parameters, no significant difference was found in translations' and rotations' effects (P = 0.13). A WM network was constructed for each subject.

### Comparisons of regional features between musicians and non-musicians

**Figure 1:**
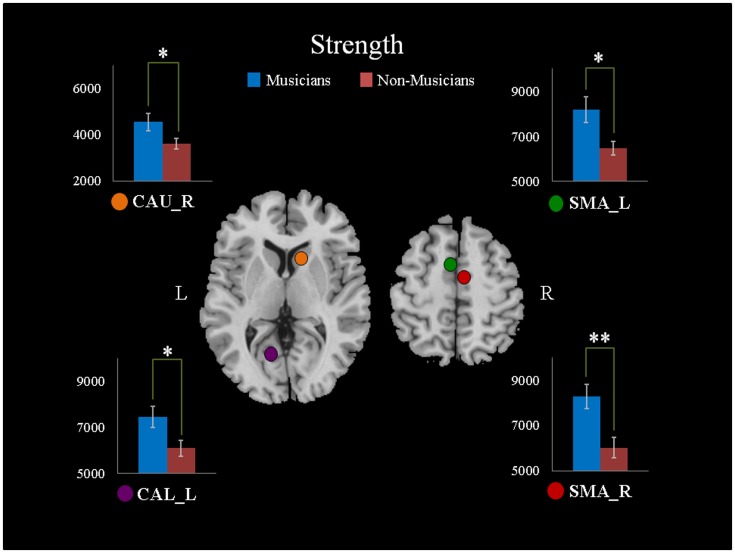
Differences in strength between the musicians and non-musicians. Significantly increased strength was observed in the bilateral supplementary motor areas (SMA_L/SMA_R), the left calcarine fissure and surrounding cortex (CAL_L) and the right caudate nucleus (CAU_R). *: P<0.05, **: P<0.01, two-tailed. L: left, R: right.

From the statistical comparison of the strength of the weighted graphs between the backbone networks of the two groups, we found that musicians exhibited significantly greater strength than the control group in the bilateral supplementary motor area (SMA) (left SMA: P = 0.014, T = 2.61; right SMA: P = 0.0035, T = 3.21), the left primary visual cortex (calcarine fissure and surrounding cortex, CAL) (P = 0.026, T = 2.35) and the right caudate nucleus (CAU) (P = 0.043, T = 2.12) (Figure. 1). The degree of each node was also compared, but no significant differences were found.

**Figure 2:**
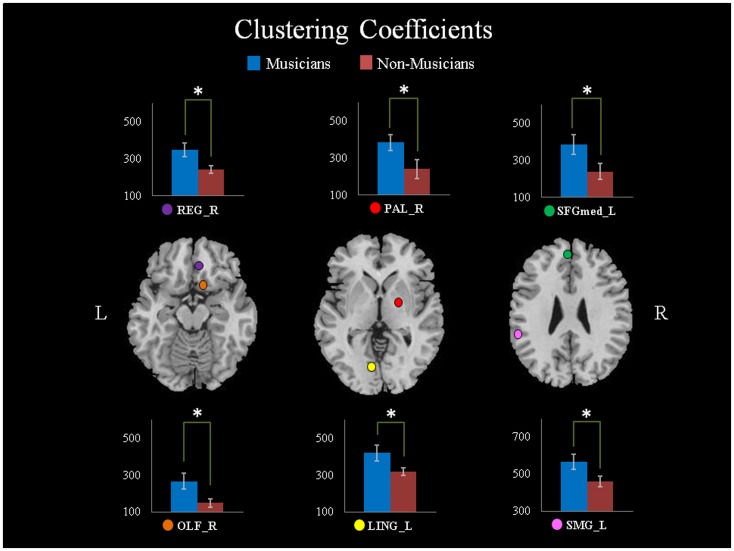
Differences in the generalized clustering coefficients of the musicians and non-musicians. Increased clustering coefficients were observed in the right olfactory cortex (OLF_R), the left medial superior frontal gyrus (SFGmed_L), the right gyrus rectus (REG_R), the left lingual gyrus (LING_L), the left supramarginal gyrus (SMG_L), and the right pallidum (PAL_R). *: P<0.05, **: P<0.01, two-tailed. L: left, R: right.

**Figure 3:**
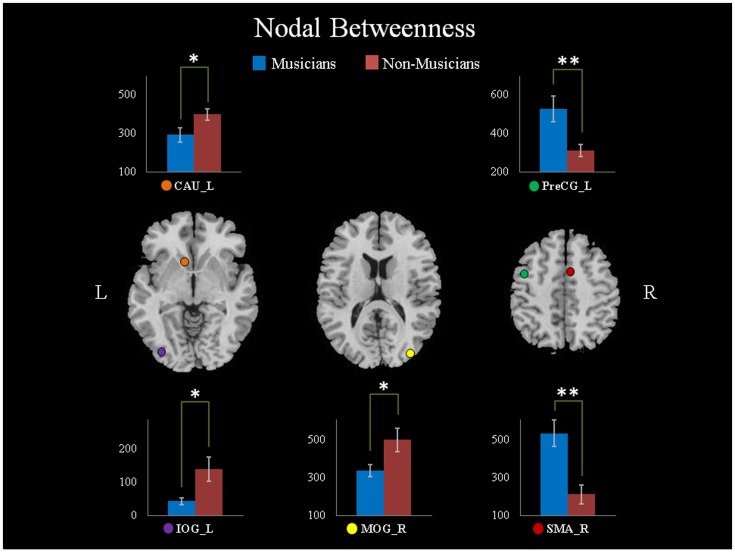
Differences in nodal betweenness between the musicians and non-musicians, including the left precentral gyrus (PreCG_L), the right supplementary motor areas (SMA_R), the right middle occipital gyrus (MOG_R), the left inferior occipital gyrus (IOG_L), and the left caudate nucleus (CAU_L). *: P<0.05, **: P<0.01, two-tailed. L: left, R: right.

For the local network parameters, we found that there were significant differences in the generalized clustering coefficients and nodal betweenness centrality. Musicians exhibited predominant enhanced generalized clustering coefficients in the right olfactory cortex (OLF) (P = 0.027, T = 2.33), the left medial superior frontal gyrus (SFGmed) (P = 0.044, T = 2.11), the right gyrus rectus (REG) (P = 0.017, T = 2.53), the left lingual gyrus (LING) (P = 0.046, T = 2.09), the left supramarginal gyrus (SMG) (P = 0.045, T = 2.10), and the right pallidum (PAL) (P = 0.042, T = 2.14) (Figure. 2). The nodal betweenness centrality in the left precentral gyrus (PreCG) (P = 0.0082, T = 2.86) and the right SMA (P = 0.00076, T = 3.81) were significantly increased in the musicians. Decreased nodal betweenness centrality was observed in the right middle occipital gyrus (MOG) (P = 0.024, T = −2.38), the left inferior occipital gyrus (IOG) (P = 0.017, T = −2.54), and the left CAU (P = 0.029, T = −2.31) (Figure. 3). A comparison of the mean shortest path length showed no significant difference between the two groups.

### Comparisons of global network features

Using graph theoretical analyses, we showed that the WM networks of both groups exhibited a much higher local efficiency and a similar global efficiency compared with the matched random networks (musicians: *E_loc_(G)/E_loc_(G_random_)* = 10.11, *E_glob_(G)/E_glob_(G_random_)* = 0.77; non-musicians: *E_loc_(G)/E_loc_(G_random_)* = 10.12, *E_glob_(G)/E_glob_(G_random_)* = 0.76). These results suggest that the WM structural networks in the two groups exhibited small-world characteristics. Furthermore, for the whole network, there were no significant differences between the two groups in clustering coefficient, mean shortest path length, global efficiency, local efficiency, and small-world attributes.

In musicians group, the correlations between the onset of musical training and the features of special brain regions mentioned in this section were also analyzed. No significant correlation between them was shown.

## Discussion

Musical training has been shown to involve several sensory, motor, auditory, visual, and emotional functions [4], [7], [8]. In the present study, we explored the changes in the WM network that were attributed to long-term musical training. The structural network topological attributes of the female musicians and the age- and gender-matched controls were assessed. In line with previous studies [23], [31], we observed alterations in the WM networks of the musicians when compared to the controls. Overall, we observed that musicians had enhanced connection strength in the motor and visual function-related regions, the basal ganglia, and the orbital part of frontal lobe, while the SMA and primary motor cortex also demonstrated increased betweenness centrality. Additionally, musicians showed improved weighted clustering coefficients at the supramarginal gyrus, which is related to language perception and processing. Small-world characteristics were identified in the WM structural networks of both groups; however, we did not observed any significant differences in the global network features of the two groups. Our findings extend our understanding of the network level WM plasticity of musicians who have had long-term musical training.

### Enhanced features of WM network in motor and visual regions

Compared to non-musicians, musicians exhibited significantly increased WM connectivity strength in the bilateral SMA, the left CAL, and the right CAU. Previous studies have shown that musical training involves several sensory, motor, auditory, visual, and emotional functions [7], [23], [53]. In some ways, musical practice is motor and sensorimotor training. On one hand, previous structural studies have shown that musicians had increased gray matter volume in several motor-related regions, including the primary motor cortex (especially the primary hand motor area) and the cerebellum [23], [54]. On the other hand, DWI studies revealed that musical practice could change the FA values in a variety of WM regions, such as the internal capsule, the right superior longitudinal fasciculus and the corticospinal tract (CST) [29], [30], [31]. Some of these regions, such as the CST, are important to convey sensorimotor information and other cognitive information [55]. Furthermore, functional differences in the activation of the SMA and other motor regions during motor tasks have been demonstrated in piano players and control subjects [3], [16], [17]. The SMA has been shown to play a critical role in self-initiated movements, movement sequences and learning, cognitive control and control of bimanual sequential finger movements [56], [57], [58], [59], [60]. Our results are consistent with previous findings, and we conclude that long-term musical training could increase the connectivity strength in the motor cortex. Additionally, the nodal betweenness centrality values increased in the primary motor cortex (PreCG) and the SMA. Nodal betweenness means the shortest paths passed a given node accounted for the proportion of all shortest paths in the network and is associated with information traffic capacity. Increased nodal betweenness might suggest that the information traffic capacity of the primary motor cortex and SMA became greater; in other words, more information was transferred through these regions. Therefore, abundant musical experience could increase information transmission for motor-related functions.

Altered nodal features were also observed in the occipital lobe, which is involved in visual processing. Additionally, the increased connection strength in the left CAL is of particular interest due to the visual information transfer in a musician's brain. This region is known to play an important role in vision and is also an important region for musical experience [61]. Functional neuroimaging studies have shown that musicians have greater activity in the CAL during musical memory tasks than non-musicians [62]. Musicians are skilled in translating visually presented musical symbols into complicated movements of the fingers and hands, as well as memorizing long musical phrases. In other words, musicians need to repetitively practice transforming visual-spatial information into motor function during their entire career; thus, we have reason to believe that long-term musical training and performance enhanced the information transfer efficiency between the motor, visual, and other regions.

Musical training leads to fine-grained perception and motor control, and excellent integration of multi-sensory systems and motor systems is a trait of musicians. Short-term training-induced changes in sensorimotor integration were observed in previous studies [63], [64]. The increased integration of these motor and sensorimotor systems was observed in musicians in our previous study using resting state fMRI [18]. In the current study, the extensive findings in the WM networks support the hypothesis that plasticity is induced in the motor and visual systems by music training. Although early music training seems to be particularly effective at increasing plasticity [65], [66], we did not find a significant relationship between the features and the subject's age at the onset of music training. The relatively small sample size might be insufficient to uncover this relationship, and its definitive elucidation will require further investigations using larger sample sets.

### Changes in local efficiency at supramarginal gyrus

The supramarginal gyrus is most likely involved in language perception and processing, and its lesions may cause receptive aphasia or transcortical sensory aphasia [67]. Recent meta-analysis also showed the left supramarginal gyrus was specifically related to processing semantics and language [68]. Here, the nodal generalized clustering coefficient (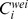), which shows how closely the nodes neighboring the *i*th node are connected to each other, was increased in the left SMG in the musicians. This result means that the musicians had closer local connectedness and higher efficiency information transfer in the subgraphs where the left SMG were the central nodes. In other words, the findings showed that the left SMG might be an important hinge of information transfer during musical experiences. The improvement of WM connectivity in the SMG might contribute to the musical training benefits observed in the neural encoding of speech and language [69].

### WM improvement at orbital frontal cortex and basal ganglia

Increased 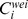 was also observed in the orbital part of the frontal lobe, including the OLF and REG, in the musicians in this study. According to previous studies [70], [71], the orbital frontal lobe is involved in emotional regulation. Blood and Zatorre also found increased regional cerebral blood flow in the orbitofrontal cortex during chills induced by music [72]. Increased 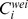 in the orbital frontal lobe might reflect the possible foundation of an emotional effect evoked by listening to music or playing music.

Moreover, musicians also exhibited increased strength in the right CAU, which is in accordance with a previous study that focused on FA values [29]. This study observed greater FA values in the central aspects of the CAU in subjects with musical training. We presumed that musical training could increase the FA values and the number of fibers in the CAU. Furthermore, a functional study focused on working memory for auditory information showed that musicians showed stronger activation in the right CAU compared to non-musicians [73]. This indirect evidence demonstrated that more information passed through the right CAU of musicians than that of non-musicians during long-term musical training. Moreover, the caudate nucleus were also sensitive to emotional content [74] and were co-activated with the orbital part of the frontal lobe in response to visual beauty [75]. The striatum is involved in musical pleasure [76], and these results encourage the exploration of the increased clustering coefficient in the pallidum. Taken together, the plasticity of the WM network features in the orbital frontal cortex and basal ganglia might implicate the emotional effects of music.

### Global features of WM network

Both groups displayed small-world characteristics, but no significant differences in the global network features, including small-world parameters, were observed between the groups in the current study. We speculate that music training might induce local plasticity, rather than change the global features of the WM networks. An alternative reason is that the musicians were healthy and did not suffer from neuropsychiatric disorders, such as schizophrenia, or lesions in the brain. It is most likely difficult to alter global features by creating new links and/or reducing quondam links between two brain regions based on existing parcellation in musicians. Therefore, we are inclined to believe that musical training may lead to some changes in the gray matter volume or in WM connectivity strength between regions, as well as other potential unknown changes.

### Methodological Issues and Limitations

Many studies revealed that there are many significant brain anatomical differences between men and women in both gray matter and WM [77], [78], [79], [80]. Thus, all of the subjects chosen for this study were female to avoid influences caused by these gender differences. However, one should exercise caution when generalizing a single gender finding for the whole population. The sample size we obtained was relatively small. In addition, our results with uncorrected p-values were preliminary and further investigations with large sample sizes are required.

We constructed the structural network using the probabilistic tractography approach on the basis of the DWI data; thus, the information about the direction of nerve fibers could not be obtained. In addition, the parameters of DWI acquisition might influence the findings. The number of orientations (n = 20 in the current study) might affect the robustness for estimating the tensor-orientation and mean diffusivity [81]. Therefore, we could not distinguish between efferent and afferent projections. Additionally, probabilistic tractography is accompanied by some open problems related to the recognition of false fiber trajectories and to the corresponding elimination of nuisance connections.

Although the atlas we used for the parcellation of gray matter was carefully created by taking into account the relevant anatomical and functional details, the use of advanced integrative atlases based on finer cytoarchitecture, myeloarchitecture, and MRI procedures may be better suited for future applications [82], [83].

Considering the post-hoc analysis that was used in this study, we can't exclude the possibility that differences between two groups could have existed already before and might be due to other factors other than musical training.

## Conclusions

In summary, our preliminary findings demonstrated the plasticity of features in the WM networks of the motor and visual systems, SMG, basal ganglia, and orbital frontal cortex in musicians. In other words, long-term musical training and performance may contribute to the improved efficiency of communication between brain regions with sensory, motor, and emotional functions. We provide evidence for the music-training-induced plasticity of the brain structural network. Combined with previous research, our findings suggest that musical training can increase gray matter volume [54], [84], change the diffusion parameter of WM [30], [31], and can also enhance WM connectivity.

Further studies focusing on musical training or other learning-related plasticity may combine these multimodal MRI techniques, and these studies would yield a more comprehensive understanding of how the brain changes during long-term training or learning.
